# Effectiveness of Mobile Health Interventions Promoting Physical Activity and Lifestyle Interventions to Reduce Cardiovascular Risk Among Individuals With Metabolic Syndrome: Systematic Review and Meta-Analysis

**DOI:** 10.2196/17790

**Published:** 2020-08-31

**Authors:** Irene Sequi-Dominguez, Celia Alvarez-Bueno, Vicente Martinez-Vizcaino, Rubén Fernandez-Rodriguez, Alicia del Saz Lara, Iván Cavero-Redondo

**Affiliations:** 1 Universidad de Castilla-La Mancha Health and Social Research Center Cuenca Spain; 2 Universidad Politécnica y Artística del Paraguay Asunción Paraguay; 3 Universidad Autónoma de Chile Facultad de Ciencias de la Salud Talca Chile; 4 Movi-Fitness SL Universidad de Castilla-La Mancha Cuenca Spain

**Keywords:** mobile health, mobile technology, telemedicine, metabolic syndrome, physical activity, lifestyle intervention, systematic review, meta-analysis

## Abstract

**Background:**

Physical activity and lifestyle interventions, such as a healthy diet, have been proven to be effective approaches to manage metabolic syndrome. However, these interventions require great commitment from patients and clinicians owing to their economic costs, time consumption, and lack of immediate results.

**Objective:**

The aim of this systematic review and meta-analysis was to analyze the effect of mobile-based health interventions for reducing cardiometabolic risk through the promotion of physical activity and healthy lifestyle behaviors.

**Methods:**

PubMed, Scopus, Web of Science, Cochrane Central Register of Controlled Trials, and SPORTdiscus databases were searched for experimental studies evaluating cardiometabolic risk indicators among individuals with metabolic syndrome who were included in technology-assisted physical activity and lifestyle interventions. Effect sizes, pooled mean changes, and their respective 95% CIs were calculated using the DerSimonian and Laird method. Outcomes included the following clinical and biochemical parameters: body composition (waist circumference [WC] and BMI), blood pressure (systolic blood pressure [SBP] and diastolic blood pressure [DBP]), glucose tolerance (fasting plasma glucose [FPG] and glycated hemoglobin A1c [HbA_1c_]), and lipid profile (total cholesterol, low-density lipoprotein cholesterol, high-density lipoprotein cholesterol [HDL-C], and triglycerides).

**Results:**

A total of nine studies were included in the meta-analysis. Owing to the scarcity of studies, only pooled mean pre-post changes in the intervention groups were estimated. Significant mean changes were observed for BMI (−1.70 kg/m2, 95% CI −3.20 to −0.20; effect size: −0.46; *P*=.03), WC (−5.77 cm, 95% CI −9.76 to −1.77; effect size: −0.54; *P*=.005), SBP (−7.33 mmHg, 95% CI −13.25 to −1.42; effect size: −0.43; *P*=.02), DBP (−3.90 mmHg, 95% CI −7.70 to −0.11; effect size: −0.44; *P*=.04), FPG (−3.65 mg/dL, 95% CI −4.79 to −2.51; effect size: −0.39; *P*<.001), and HDL-C (4.19 mg/dL, 95% CI 2.43-5.95; effect size: 0.23; *P*<.001).

**Conclusions:**

Overall, mobile-based health interventions aimed at promoting physical activity and healthy lifestyle changes had a strong positive effect on cardiometabolic risk indicators among individuals with metabolic syndrome. Nevertheless, further research is required to compare this approach with usual care in order to support the incorporation of these technologies in health systems.

**Trial Registration:**

PROSPERO CRD42019125461; https://tinyurl.com/y3t4wog4.

## Introduction

Metabolic syndrome (MetS) is a cluster of cardiometabolic risk factors that include abdominal obesity, dyslipidemia, hypertension, and insulin resistance [1,2]. MetS has become a worldwide epidemic in parallel with the increase in unhealthy behaviors, such as high rates of physical inactivity and energy dense diets, which have led to alarming obesity prevalence rates in wealthy countries, as well as in developing countries, but to a lesser extent [3]. MetS increases the risk of diabetes mellitus and cardiovascular disease (CVD) in patients with or without a history of cardiovascular events [4]; thus, its early detection may be an important strategy to improve patients’ future cardiometabolic risk.

Traditionally, MetS has not been clinically addressed as a single entity but has been managed by treating each of its individual components separately by recommending lifestyle changes (healthy diet and exercise) and pharmacological or even surgical approaches (specifically bariatric surgery, when required). Physical activity interventions have been proven to be effective in reducing CVD risk factors by increasing cardiorespiratory fitness, and dietary interventions have been proven to be effective in decreasing adiposity [5]. In addition, physical activity interventions have been shown to be effective at 12 weeks or more for cardiometabolic parameters [6]. So far, randomized controlled trials (RCTs) of these interventions have required intensive one-on-one or group lifestyle recommendations, raising questions about the feasibility and scalability of implementing these interventions outside of research settings [7].

Mobile-based health (mHealth) technologies can be conceptualized as the remote delivery of health care and exchange of health care information [8]. These technologies can be seen as a complement for some traditional health care methods that, by enabling remote health consultations and monitoring, improve accessibility to health services and the efficiency of some health interventions [8]. Since mobile apps play a key role in everyday life, lifestyle interventions based on these technologies may increase the potential for scalability of interventions and improve their long-term effects and sustainability. In fact, it is expected that the prevention and management of the most common health disorders, which traditionally place a large burden on personnel and resources, will gradually shift to a disease management model in the near future, introducing the use of mHealth [9].

Thus, the aim of this systematic review and meta-analysis was to analyze the effect of lifestyle interventions, including physical activity recommendations through mHealth technologies, on CVD risk factors among individuals with MetS.

## Methods

### Design

This systematic review and meta-analysis was registered in PROSPERO (registration number: CRD42019125461) and was reported following the Preferred Reporting Items for Systematic Reviews and Meta-Analyses (PRISMA) guidelines [10]. The recommendations of the Cochrane Handbook for Systematic Reviews of Interventions [11] were followed to conduct this systematic review and meta-analysis.

### Search Strategy

**Figure 1:**
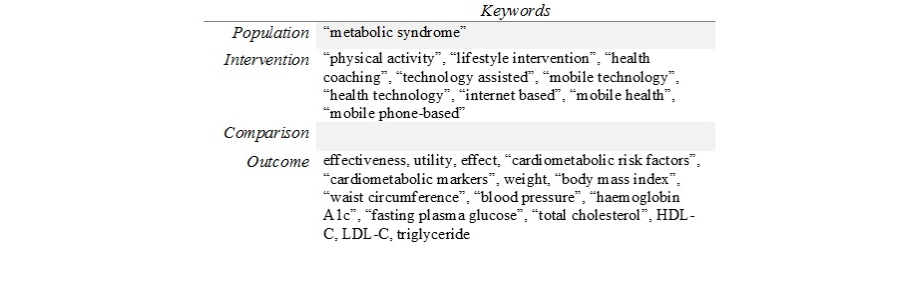
PICO (population, intervention, comparison, and outcome) search strategy.

PubMed (via Medline), EMBASE (via Scopus), Web of Science, Cochrane Central Register of Controlled Trials, and SPORTdiscus databases were searched from their inception to August 2019 following the same PICO (population, intervention, comparison, and outcome) strategy (Figure 1) that included the following: ((“metabolic syndrome”) AND (“physical activity” OR “lifestyle intervention” OR “health coaching” OR “technology assisted” OR “mobile technology” OR “health technology” OR “internet based” OR “mobile health” OR “mobile phone-based”) AND (effectiveness OR utility OR effect OR “cardiometabolic risk factors” OR “cardio-metabolic markers” OR weight OR “body mass index” OR “waist circumference” OR “blood pressure” OR “hemoglobin A1c” OR “fasting plasma glucose” OR “total cholesterol” OR HDL-C OR LDL-C OR triglyceride)).

### Selection of Studies

Eligible articles were experimental studies (RCTs or nonrandomized experimental studies and single-arm pre-post studies), which aimed to measure the effectiveness of lifestyle and physical activity recommendations, using mHealth technologies to reduce cardiometabolic risk factors in individuals with MetS. Studies not written in English or Spanish, including patients with diabetes, or not reporting pre- and postcardiometabolic risk factor values were excluded.

Interventions were classified according to their main characteristics as follows: (1) performing data monitoring or not; (2) carrying out lifestyle and/or physical activity recommendations; and (3) including goal setting tools or not. Outcomes were measured as mean changes in the following cardiometabolic risk indicators: body composition (BMI and waist circumference [WC]), blood pressure (systolic blood pressure [SBP] and diastolic blood pressure [DBP]), glucose tolerance (fasting plasma glucose [FPG] and glycated hemoglobin A1c [HbA_1c_]), and lipid profile (total cholesterol, high-density lipoprotein cholesterol [HDL-C], low-density lipoprotein cholesterol [LDL-C], and triglycerides).

The literature search was independently conducted by two reviewers (ISD and ICR), and disagreements were solved by consensus or discussion with a third researcher (CAB).

### Data Extraction and Quality Assessment

The following information was extracted from the included studies: (1) year of publication, (2) country, (3) type of study, (4) sample characteristics (sample size and mean age), (5) intervention characteristics (design and length of intervention), and (6) MetS indicators.

The Cochrane Collaborations tool was used for assessing risk of bias in randomized trials [12], which scores six domains as low, high, or unclear risk. The Quality Assessment Tool for Quantitative Studies [13] was used for nonrandomized experimental and single-arm pre-post studies. It consists of seven domains of risk of bias that are rated as strong, moderate, or weak. Both tools assessed the risk of bias of each study as low (with no high/weak ratings), moderate (with one high/weak rating), or high (with two or more high/weak ratings) [14].

Data extraction and quality assessment were independently performed by two reviewers (ISD and ICR), and inconsistencies were solved by consensus or discussion with a third researcher (CAB). The agreement rate between reviewers was calculated using the kappa statistic.

### Statistical Analysis

The DerSimonian and Laird method [15] was used to compute the pooled mean change estimates for BMI, WC, SBP, DBP, FPG, HbA_1c_, total cholesterol, HDL-C, LDL-C, and triglycerides, with their respective 95% CIs. Because of the scarcity of RCTs, in which the difference in change between intervention and control groups for the outcome variable was calculated, we calculated the pooled mean pre-post change in the outcome variable for all the interventions (not for the control group). In multiarm trials (two or more intervention groups), we calculated separately the pooled mean pre-post change in each arm, and the common control group was not included in the analysis. Additionally, standardized mean difference scores for the pooled mean change estimates were calculated using the effect size of Cohen *d*, in which the effect was considered weak for values around 0.2, moderate for values around 0.5, strong for values around 0.8, and very strong for values greater than 1.0. When studies reported pre- and postmean values, effect size estimates were calculated for each parameter.

The heterogeneity of results across studies was evaluated using the *I^2^* statistic [16]. *I^2^* values were assessed as follows: 0%-30%, might not be important; 30%-50%, moderate heterogeneity; 50%-75%, substantial heterogeneity; and 75%-100%, considerable heterogeneity. The corresponding *P* values were also taken into account [11].

Sensitivity analyses were conducted to assess the robustness of the summary estimates and to detect if any particular study accounted for a large proportion of heterogeneity. Random-effects meta-regression models were used to evaluate whether pooled estimates were influenced by the mean age of participants and the percentage of women [17]. Finally, publication bias was evaluated through visual inspection of funnel plots, as well as using the method proposed by Egger [18].

The significance value of the pooled mean change was estimated based on the 95% CI. Statistical analyses were performed using STATA SE software, version 15 (StataCorp).

## Results

### Systematic Review

**Figure 2:**
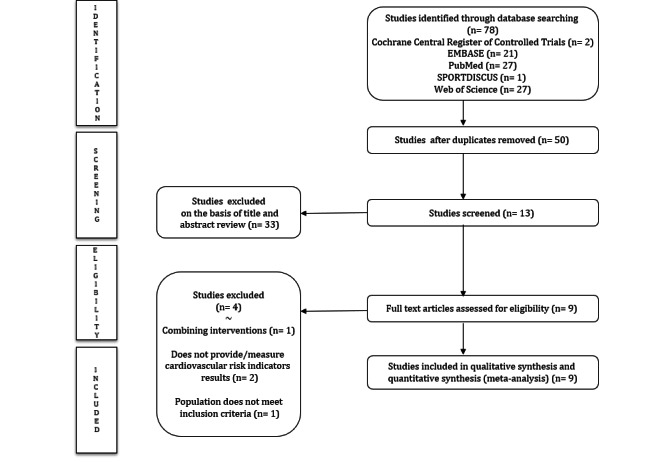
Preferred Reporting Items for Systematic Reviews and Meta-Analyses (PRISMA) diagram.

After removing duplicate studies, a total of 47 articles were selected for full-text review following title and abstract screening. Finally, nine studies [19-27] were included in this systematic review (Figure 2).

Of the included studies, five were RCTs [19,23-26] and four were single-arm pre-post studies [20-22,27]. Studies were published between 2013 and 2018, and conducted in four different countries (two in Canada [26,27], one in Germany [23], three in the Republic of Korea [21,22,25], and three in the United States [19,20,24]).

The sample size of the included studies ranged from 12 to 421 participants (51.7% females, although two studies included men only [21,22]), and the mean age varied between 38.4 and 59.7 years. All participants met the diagnostic criteria for MetS (according to the Adult Treatment Panel III guidelines or the International Diabetes Federation) and were able to access and use the technology required for each intervention.

The interventions were mainly based on physical activity and lifestyle recommendations, with personalization in some cases [20-23], and were delivered through a website, videoconferencing, or an app. The effects of the recommendations were assessed using telemonitoring through mobile devices. In three of the included studies, the interventions were strengthened using self-goal setting tools such as a behavioral strategy for patients to help them visualize their accomplishments and objectives [24,26,27]. The duration of interventions ranged from 8 to 48 weeks, with the number of clinical encounters varying between 2 and 24, and most of them were in-person encounters to perform periodic clinical evaluations (Table 1).

**Table 1:** Characteristics of the included studies.

First author, year of publication	Country	Study design	Intervention characteristics						
			Sample size (n [%] female)	Mean age (years)	Intervention design	Number of clinical encounters	Duration	Cardiometabolic risk outcomes	
Azar et al, 2016 [19]	USA	RCT^a^	n=74 (44 [59%]) IG^b^: n=37 CG^c^: n=37	59.7 (SD 11.2)	IG: Data monitoring, PA^d^ and lifestyle web advice, and weekly videoconferencing CG: Intervention 3 months delayed	24 virtual group sessions Seven in-person PA sessions	24 weeks	Weight, weight change, BMI, WC^e^, SBP^f^, DBP^g^, TC^h^, HDL-C^i^, LDL-C^j^, TC/HDL ratio, and triglyceride	
Everett et al, 2018 [20]	USA	Pre-post study	n=38 (24 [63%])	57.2 (SD 9.1)	Data monitoring and PA, weight reduction, and diet personalized advice through a smartphone	Two face-to-face sessions	12 weeks	Weight, percentage weight change, BMI, WC, SBP, DBP, HbA_1c_^k^, and FPG^l^	
Kim and Kang, 2013 [21]	Republic of Korea	Pre-post study	n=18 (0 [0%])	43.1 (SD 7.4)	PA and weight control personalized advice through a website and SMS text messages	Weekly web visits	8 weeks	Weight, visceral fat mass, WC, SBP, DBP, HDL-C, TG, FPG, and CVD^m^ risk	
Kim et al, 2014 [22]	Republic of Korea	Pre-post study	n=48 (0 [0%]) IG: n=24 CG: n=24	IG: 40.88 (SD 7.70) CG: 38.38 (SD 6.82)	PA and weight control personalized advice through a website and SMS text messages	Weekly online sessions	16 weeks	Weight, body fat, VFM^n^, WC, SBP, DBP, HDL-C, TG, FPG, and CVD risk	
Luley et al, 2014 [23]	Germany	RCT	n=178 (73 [41%]) IG1: n=60 (18 [30%]) IG2: n=58 (27 [47%]) CG: n=60 (28 [47%])	IG1: 50.3 (SD 7.8) IG2: 50.3 (SD 8.0) CG: 50.1 (SD 8.1)	IG1: PA and diet recommendations, data telemonitoring, and weekly feedback letters IG2: PA and diet recommendations, data telemonitoring, and monthly feedback calls CG: PA and diet in-person recommendations	Four in-person sessions	48 weeks	Weight loss; BMI, WC, SBP, DBP, TC, HDL-C, LDL-C, TG, apolipoprotein B, uric acid, alanine aminotransferase, aspartate aminotransferase, high-sensitivity CRP^o^, FPG, HbA_1c_, and HOMA-IR^p^	
Mann et al, 2016 [24]	USA	RCT	n=54 (45 [83%]) IG: n=27 CG: n=27	IG: 47.5 (SD 11.99) CG: 43.67 (SD 9.28)	IG: Data monitoring, PA and diet recommendations, and goal setting using electronic medical records CG: Traditional recommendations and follow-up	Two compulsory in-person sessions	24 weeks	Weight, BMI, TC, HCL-C, LDL-C, TG, HbA_1c_, REAP-S^q^ score, risk knowledge, risk perception, total step average, and 7-day step average	
Oh et al, 2015 [25]	Republic of Korea	RCT	IG: n=212 (113 [53%]) CG: n=209 (99 [47%])	IG: 46.78 (SD 13.11) CG: 50.35 (SD 14.24)	IG: Body composition and pedometer data remote monitoring, and personalized PA and health online advice CG: Data records and PA and diet recommendations	Four in-person sessions	24 weeks	Weight and BMI	
Petrella et al, 2014 [26]	Canada	RCT	IG: n=75 (55 [73%]) CG: n=74 (56 [76%])	IG: 55.7 (SD 10.1) CG: 57.8 (SD 8.7)	IG: Data telemonitoring, PA prescription, and goal setting CG: PA prescription and goal setting	Four in-person sessions	12 weeks	WC, SBP, DBP, TC, HDL-C, LDL-C, TG, FPG, HbA_1c_, HOMA-IR, and high-sensitivity CRP	
Stuckey et al, 2013 [27]	Canada	Pre-post study	n=12 (9 [75%])	56.9 (SD 7.0)	PA prescription, goal setting, and data telemonitoring	Two in-person sessions	8 weeks	WC, SBP, DBP, TG, HDL-C, FPG, VO_2_ max^r^, and steps	

^a^RCT: randomized controlled trial.

^b^IG: intervention group.

^c^CG: control group.

^d^PA: physical activity.

^e^WC: waist circumference.

^f^SBP: systolic blood pressure.

^g^DBP: diastolic blood pressure.

^h^TC: total cholesterol.

^i^HDL-C: high-density lipoprotein cholesterol.

^j^LDL-C: low-density lipoprotein cholesterol.

^k^HbA_1c_: glycated hemoglobin A_1c_.

^l^FPG: fasting plasma glucose.

^m^CVD: cardiovascular disease.

^n^VFM: visceral fat mass.

^o^CRP: C-reactive protein.

^p^HOMA-IR: homeostatic model assessment of insulin resistance.

^q^REAP-S: rapid eating and activity assessment for patients.

^r^VO_2_ max: predicted maximal oxygen capacity.

### Risk of Bias

Seven out of nine studies were assessed as having a high risk of bias (including all single-arm pre-post studies), and the other two were assessed as having a moderate risk of bias. Analyzing each study individually, all single-arm pre-post studies had the lowest scores in the confounders and blinding domains (Table 2). All RCTs had a high risk of bias in the performance and detection bias domains (Table 3).

**Table 2:** Quality assessment of the included pre-post studies.

First author, year of publication	Selection bias	Study design	Confounders	Blinding	Data collection	Withdrawals	Risk of bias
Everett et al, 2018 [20]	Moderate	Moderate	Weak	Weak	Weak	Strong	High
Kim and Kang, 2013 [21]	Moderate	Moderate	Weak	Weak	Strong	Strong	High
Kim et al, 2014 [22]	Strong	Moderate	Weak	Weak	Strong	Strong	High
Stuckey et al, 2013 [27]	Moderate	Weak	Weak	Weak	Strong	Strong	High

**Table 3:** Quality assessment of the included randomized controlled trials.

First author, year of publication	Selection bias	Performance bias	Detection bias	Attrition bias	Reporting bias	Other bias	Risk of bias
Azar et al, 2016 [19]	Low	High	Unclear	Low	Low	Low	Moderate
Luley et al, 2014 [23]	Unclear	High	High	Low	Unclear	Low	High
Mann et al, 2016 [24]	High	High	High	Low	Low	Unclear	High
Oh et al, 2015 [25]	Low	Unclear	Unclear	Low	Low	Low	Low
Petrella et al, 2014 [26]	High	High	High	Low	Low	Unclear	High

### Meta-Analysis

Because of the small number of RCTs, only pooled effect estimates were calculated for mHealth promoting physical activity and lifestyle interventions in pre-post studies. The pre-post pooled mean changes with their heterogeneity statistics for each outcome category are presented below.

#### Body Composition

**Figure 3:**
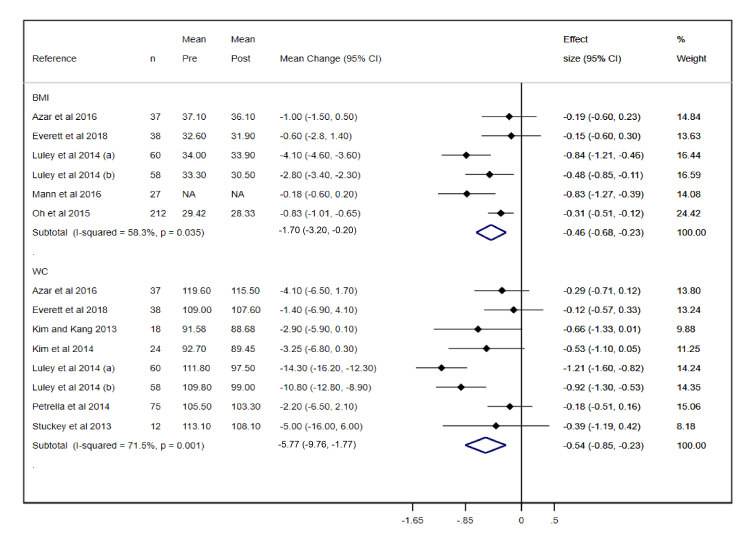
Forest plot of meta-analysis of mean changes and effect sizes for body composition parameters. WC: waist circumference.

The mean changes were −1.70 kg/m^2^ (95% CI −3.20 to −0.20; effect size: −0.46) for BMI and −5.77 cm (95% CI −9.76 to −1.77; effect size: −0.54) for WC. All pooled estimates showed moderate to substantial heterogeneity (BMI: *I^2^*=58.3%; WC: *I^2^*=71.5%) (Figure 3).

#### Blood Pressure

**Figure 4:**
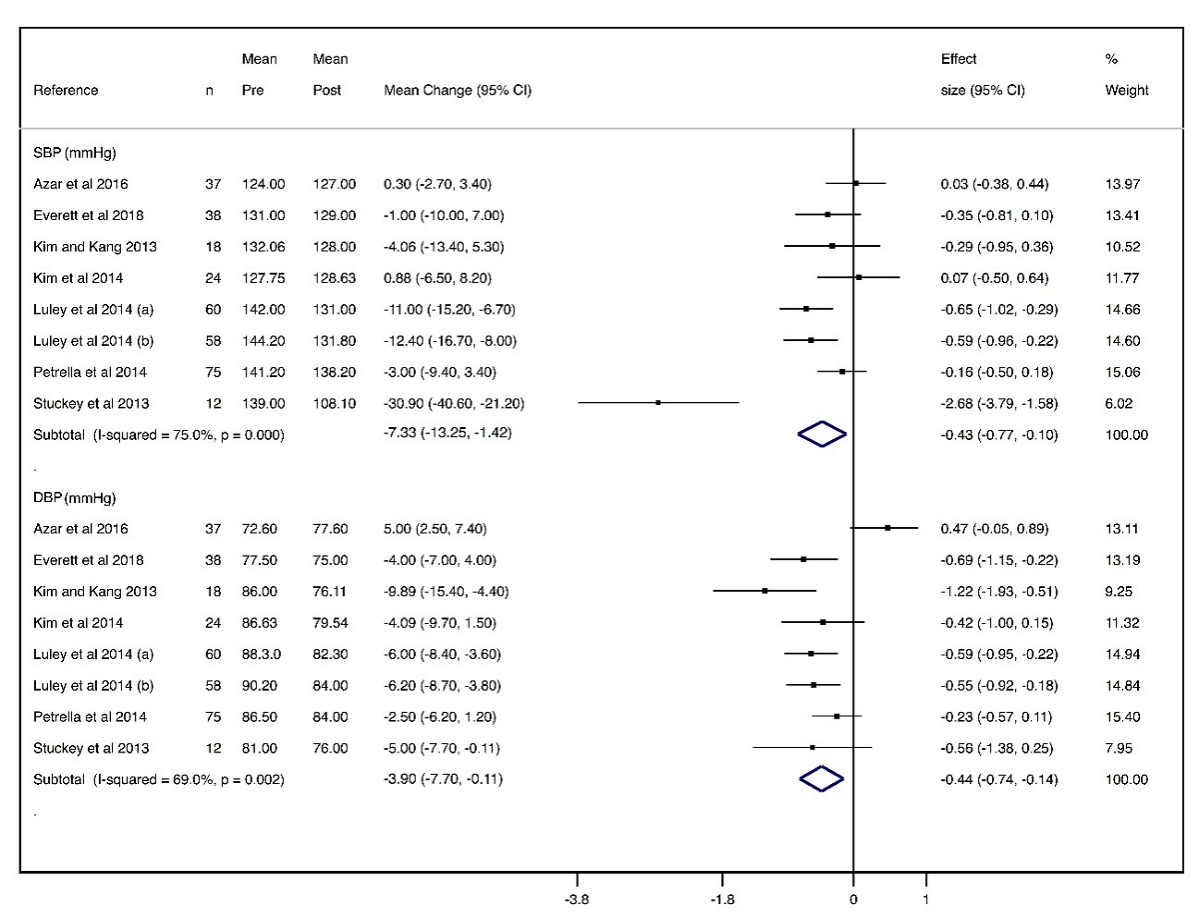
Forest plot of meta-analysis of mean changes and effect sizes for blood pressure parameters. DBP: diastolic blood pressure; SBP: systolic blood pressure.

The mean changes were −7.33 mmHg (95% CI −13.25 to −1.42; effect size: −0.43) for SBP and −3.90 mmHg (95% CI −7.70 to −0.11; effect size: −0.44) for DBP, with substantial heterogeneity for SBP (*I^2^*=75%) and DBP (*I^2^*=69%) (Figure 4).

#### Glucose Tolerance

**Figure 5:**
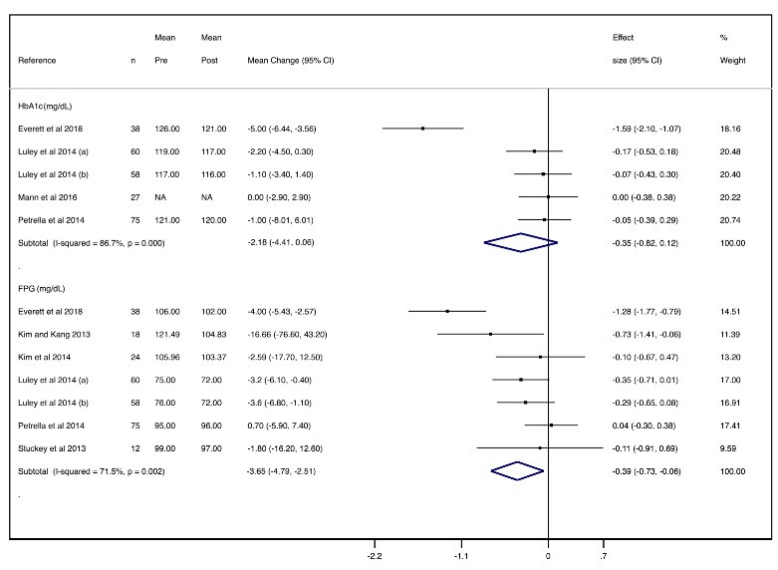
Forest plot of meta-analysis of mean changes and effect sizes for glucose tolerance parameters. FPG: fasting plasma glucose; HbA_1c_: glycated hemoglobin A_1c_.

The mean changes were −2.18 mg/dL (95% CI −4.41 to 0.06; effect size: −0.35) for HbA_1c_, with considerable heterogeneity (*I^2^*=86.7%), and −3.65 mg/dL (95% CI −4.79 to −2.51; effect size: −0.39) for FPG, with substantial heterogeneity (*I^2^*=71.5%) (Figure 5).

#### Lipid Profile

**Figure 6:**
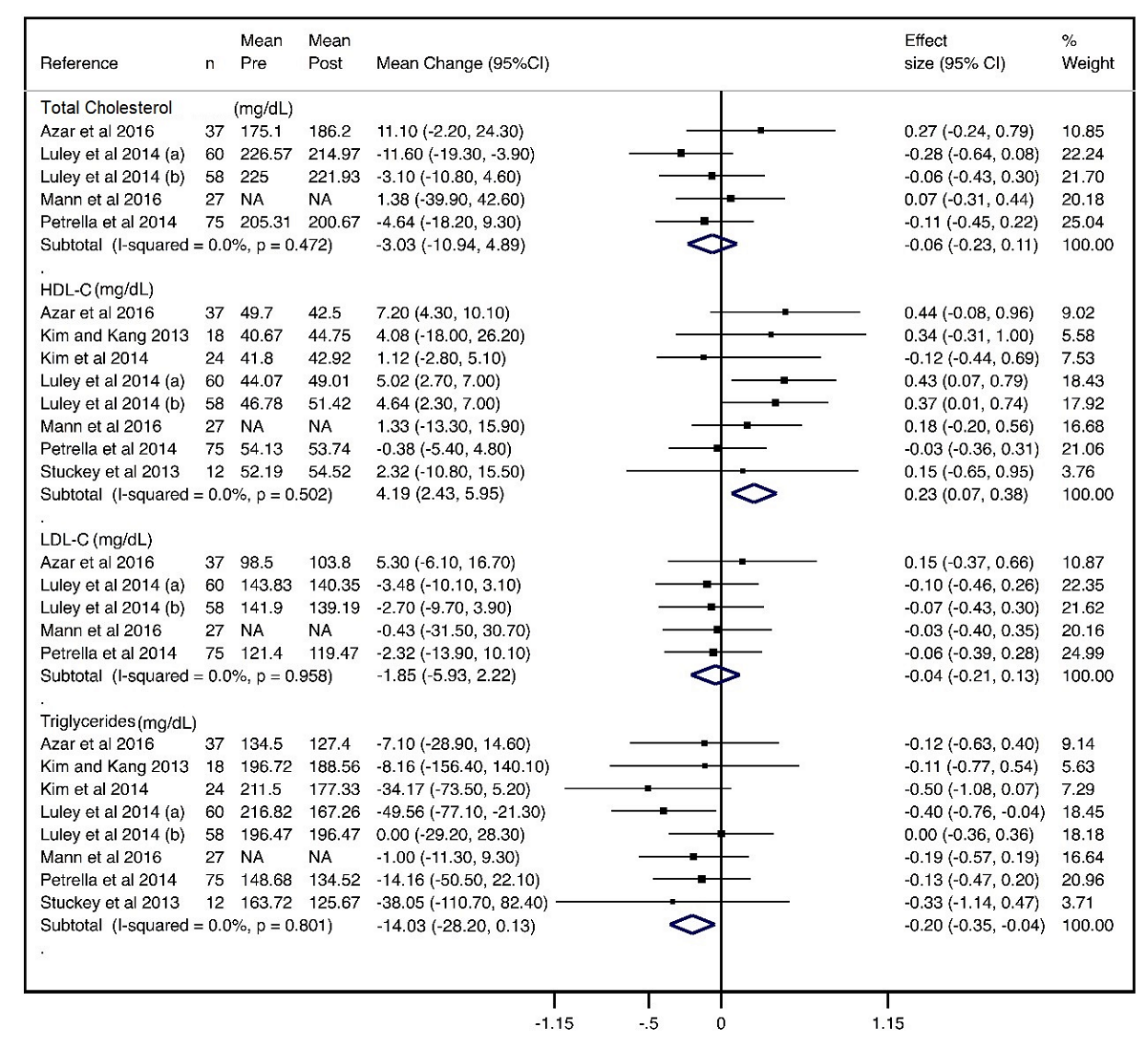
Forest plot of meta-analysis of mean changes and effect sizes for lipid profile parameters. HDL-C: high-density lipoprotein cholesterol; LDL-C: low-density lipoprotein cholesterol.

The mean changes were −3.03 mg/dL (95% CI −10.94 to 4.89; effect size: −0.06) for total cholesterol, with no heterogeneity (*I^2^*=0.0%), −1.85 mg/dL (95% CI −5.93 to 2.22; effect size: −0.04) for LDL-C, with no heterogeneity (*I^2^*=0.0%), and −14.03 mg/dL (95% CI −28.20 to 0.13; effect size: −0.20) for triglycerides, with no heterogeneity (*I^2^*=0.0%). Pooled mean changes were not relevant for any of the lipid parameters, except for HDL-C, which increased 4.19 mg/dL (95% CI 2.43-5.95; effect size: 0.23), with no heterogeneity (*I^2^*=0.0%) (Figure 6).

### Sensitivity Analysis

Studies were removed from the analysis one at a time in order to examine their individual impact on the pooled estimates. The pooled mean change of FPG was only significantly modified after removing the study by Everett et al [20] (−3.04 mg/dL; 95% CI −4.94 to −1.15; *P*=.002). None of the remaining studies potentially modified the pooled mean change estimate in magnitude or direction.

### Meta-Regression

The random-effects meta-regression models showed that the percentage of females included in the study could influence the pooled estimates of the effect on BMI (*P*=.01) and triglycerides (*P*=.03), and the follow-up period could influence the pooled estimates of the effect on WC (*P*=.005) (Table 4).

**Table 4:** Meta-regression findings.

Variable		Age			Percentage of women					Follow-up period		
		Value, n	β (95% CI)	*P* value		Value, n	β (95% CI)	*P* value	Value, n		β (95% CI)	*P* value
**Body composition**												
	BMI (kg/m^2^)	6	0.04 (−0.37 to 0.45)	.81		6	0.07 (0.03 to 0.12)	.01	6		−0.01 (−0.03 to 0.01)	.21
	Waist circumference (cm)	8	0.06 (−0.68 to 0.80)	.86		8	0.01 (−0.16 to 0.18)	.85	8		−0.02 (−0.03 to 0.01)	.005
**Blood pressure**												
	SBP^a^ (mmHg)	8	−0.28 (−1.76 to 1.19)	.65		8	−0.12 (−0.44 to 0.21)	.41	8		0.00 (−0.04 to 0.04)	.92
	DBP^b^ (mmHg)		0.49 (−0.02 to 1.01)	.057		8	0.08 (−0.06 to 0.22)	.23	8		0.00 (−0.02 to 0.02)	.87
**Glucose tolerance**												
	HbA_1c_^c^ (mg/dL)	5	−0.29 (−1.10 to 0.52)	.34		5	0.04 (−0.08 to 0.16)	.37	5		0.01 (−0.04 to 0.07)	.449
	FPG^d^ (mg/dL)	7	0.33 (−0.12 to 0.78)	.12		7	0.08 (−0.04 to 0.19)	.42	7		0.04 (−0.02 to 0.03)	.75
**Lipid profile**												
	Total cholesterol (mg/dL)	5	1.58 (−1.10 to 4.26)	.16		5	0.28 (−0.44 to 1.00)	.31	5		0.00 (−0.02 to 0.02)	.52
	HDL-C^e^ (mg/dL)	8	0.24 (−0.08 to 0.56)	.12		8	0.01 (−0.11 to 0.13)	.80	8		0.00 (0.00 to 0.00)	.11
	LDL-C^f^ (mg/dL)	5	0.73 (−1.21 to 2.67)	.32		5	0.10 (−0.34 to 0.53)	.52	5		0.00 (−0.01 to 0.01)	.75
	Triglyceride (mg/dL)	8	0.74 (−2.97 to 4.45)	.64		8	0.49 (0.05 to 0.94)	.03	8		0.00 (−0.01 to 0.01)	.99

^a^SBP: systolic blood pressure.

^b^DBP: diastolic blood pressure.

^c^HbA_1c_: glycated hemoglobin A_1c_.

^d^FPG: fasting plasma glucose.

^e^HDL-C: high-density lipoprotein cholesterol.

^f^LDL-C: low density lipoprotein- cholesterol.

### Publication Bias

After visually examining the funnel plots and performing Egger tests for every parameter (Table 5), publication bias was only significant for WC (*P*=.04).

**Table 5:** Egger test findings.

Variable			*P* value	
**Body composition**				
	BMI (kg/m^2^)	.98		
	Waist circumference (cm)	.04		
**Blood pressure**				
	SBP^a^ (mmHg)	.45		
	DBP^b^ (mmHg)	.58		
**Glucose tolerance**				
	HbA_1c_^c^ (mg/dL)	.42		
	FPG^d^ (mg/dL)	.53		
**Lipid profile**				
	Total cholesterol (mg/dL)	.47		
	HDL-C^e^ (mg/dL)	.31		
	LDL-C^f^ (mg/dL)	.42		
	Triglyceride (mg/dL)	.24		

^a^SBP: systolic blood pressure.

^b^DBP: diastolic blood pressure.

^c^HbA_1c_: hemoglobin A_1c_.

^d^FPG: fasting plasma glucose.

^e^HDL-C: high-density lipoprotein cholesterol.

^f^LDL-C: low density lipoprotein- cholesterol.

## Discussion

### Principal Findings

Traditional approaches, such as physical activity programs, brief recommendation interventions, and pharmacological treatments, have been proven to be effective for controlling MetS [28]. However, they are expensive and time-consuming strategies that require a great commitment by both patients and practitioners. Our systematic review and meta-analysis suggested that physical activity and lifestyle interventions based on mHealth technologies are effective for reducing cardiometabolic risk, since they greatly improve body composition, blood pressure, FPG, and HDL-C levels. However, no relevant changes were observed in HbA_1c_, total cholesterol, LDL-C, or triglyceride levels.

Our findings are in line with previous evidence on mHealth interventions in chronic disease patients that reported small to moderate positive effects on primary outcomes, such as cholesterol, weight, and blood pressure [29]. These findings show similar effects both when combining mHealth interventions with usual care (consisting of regular hospital visits, regular visits by primary health care providers at home, or visits to the general practitioner) [30-35] and when mHealth interventions are carried out instead of usual care [36-40]. Such results are consistent with our findings despite the different populations targeted; however, our results show much smaller effect sizes for total cholesterol, LDL-C, and triglycerides, which may be explained by the fewer number of included studies reporting those outcomes.

Among the factors involved in the worldwide increase in sedentary behavior, the use of information and communication technologies and particularly the increase in screen time have been described as the main drivers of low daily energy expenditure [41]. Thus, to involve these technologies as vehicles of preventive interventions could be both a risk and an opportunity. Even though we were unable to demonstrate the superiority of mHealth promoting physical activity and lifestyle interventions over usual care (in-person consultations with clinicians) owing to the scarcity of studies comparing data between control and intervention groups, our results showed that mHealth interventions are effective in improving cardiometabolic risk. Our data regarding the effects of interventions based on mHealth technologies are similar to those involving traditional care [42], suggesting that they could represent an alternative treatment strategy because of their acceptability, scalability, cost-effectiveness, customization, and ability to send time-sensitive messages with an “always on” device [43]. Moreover, mHealth physical activity interventions reduce in-person health provider time and increase self-care by enabling patients to manage their progress [23].

However, our results must be interpreted cautiously, since they are threatened by several limitations that should be acknowledged. First, although a systematic search was carried out through the most well-known databases by two different researchers, some scientific contributions reported as grey literature may have been missed in our systematic search. Second, overall, the risk of bias of the included studies was rated as high. Third, there has been some criticism about using single-group studies for evaluating effectiveness [44], and only pre-post estimates for the intervention group could be used because of the scarcity of RCTs reporting the necessary data for control groups. Fourth, there was heterogeneity of interventions owing to differences in components (ie, self-monitoring, type, and persuasiveness of advice), length, and lack of precision in descriptions of the type and intensity of physical activity. Fifth, it was difficult to elucidate whether the outcome changes were due to physical activity or other lifestyle interventions as they were all designed as multicomponent interventions, and hence, it was impossible to isolate each component effect. Sixth, although our results were calculated as pre-post effect sizes, previous literature has recommended avoiding them in meta-analyses [45]. Seventh, the small sample size of some of the included studies diminished their reliability. Eighth, cardiovascular risk parameters were not the main outcomes of most studies. Lastly, none of the included studies used the mHealth evidence reporting and assessment (mERA) checklist, a tool developed by the WHO mHealth Technical Evidence Review Group in order to improve the completeness of reporting mHealth interventions [46]. Despite all of these limitations, our study, as the only updated synthesis evaluating mHealth technologies promoting physical activity and lifestyle interventions to reduce cardiovascular risk in individuals with MetS, establishes a base for future research providing more consistent evidence of their effectiveness.

### Conclusion

Our results show an overall positive effect of physical activity and lifestyle interventions delivered through mobile technologies on MetS indicators, suggesting that they may be effective tools for MetS management. However, further research is needed in order to enable a comparison between the traditional clinical approach and new interventions through mHealth technologies, as these results may be due to the lack of appropriate comparable RCTs because these technologies are novel. Additionally, estimating the independent effect of each component of these interventions would be interesting, and it is important to standardize the implementation of multicomponent interventions in such a way that enough evidence is available for consideration in clinical practice guidelines.

## Abbreviations

DBP: diastolic blood pressure
FPG: fasting plasma glucose
HbA_1c_: glycated hemoglobin A_1c_
HDL-C: high-density lipoprotein cholesterol
LDL-C: low-density lipoprotein cholesterol
MetS: metabolic syndrome
mHealth: mobile-based health
RCT: randomized controlled trial
SBP: systolic blood pressure
WC: waist circumference
